# Impact of periconceptional and preimplantation undernutrition on factors regulating myogenesis and protein synthesis in muscle of singleton and twin fetal sheep

**DOI:** 10.14814/phy2.12495

**Published:** 2015-08-11

**Authors:** Shervi Lie, Janna L Morrison, Olivia Williams-Wyss, Catherine M Suter, David T Humphreys, Susan E Ozanne, Song Zhang, Severence M MacLaughlin, David O Kleemann, Simon K Walker, Claire T Roberts, I Caroline McMillen

**Affiliations:** 1 Sansom Institute for Health Research, School of Pharmacy and Medical Sciences, University of South AustraliaAdelaide, South Australia, Australia; 2 Discipline of Physiology, School of Medical Sciences, University of AdelaideAdelaide, South Australia, Australia; 3 Victor Chang Cardiac Research InstituteDarlinghurst, New South Wales, Australia; 4 Faculty of Medicine, University of New South WalesKensington, New South Wales, Australia; 5 Metabolic Research Laboratories, Institute of Metabolic Science, Addenbrooke’s Hospital, University of CambridgeCambridge, UK; 6 South Australian Research and Development Institute, Turretfield Research CentreRosedale, South Australia, Australia; 7 Discipline of Obstetrics and Gynaecology, University of AdelaideAdelaide, South Australia, Australia; 8 The Chancellery, University of NewcastleNewcastle, New South Wales, Australia

**Keywords:** Embryo, fetus, nutrition, oocyte

## Abstract

In this study, we determined the effect of maternal undernutrition in the periconceptional (PCUN: ∼80 days before to 6 days after conception) and preimplantation (PIUN: 0–6 days after conception) periods on the mRNA and protein abundance of key factors regulating myogenesis and protein synthesis, and on the relationship between the abundance of these factors and specific microRNA expression in the quadriceps muscle of singleton and twin fetal sheep at 135–138 days of gestation. PCUN and PIUN resulted in a decrease in the protein abundance of MYF5, a factor which determines the myogenic lineage, in singletons and twins. Interestingly, there was a concomitant increase in insulin-like growth factor-1 mRNA expression, a decrease in the protein abundance of the myogenic inhibitor, myostatin (MSTN), and an increase in the mRNA and protein abundance of the MSTN inhibitor, follistatin (FST), in the PCUN and PIUN groups in both singletons and twins. These promyogenic changes may compensate for the decrease in MYF5 protein abundance evoked by early embryonic undernutrition. PCUN and PIUN also increased the protein abundance of phosphorylated eukaryotic translation initiation factor binding protein 1 (EIF4EBP1; T70 and S65) in fetal muscle in singletons and twins. There was a significant inverse relationship between the expression of miR-30a-5p, miR-30d-5p, miR-27b-3p, miR106b-5p, and miR-376b and the protein abundance of mechanistic target of rapamycin (MTOR), FST, or MYF5 in singletons or twins. In particular, the expression of miR-30a-5p was increased and MYF5 protein abundance was decreased, in PCUN and PIUN twins supporting the conclusion that the impact of PCUN and PIUN is predominantly on the embryo.

## Introduction

A series of epidemiological and experimental studies have demonstrated that exposure to poor maternal nutrition during oocyte, embryonic, or fetal development results in an increased risk of poor metabolic outcomes including insulin resistance and glucose intolerance in later life (Joshi et al. 2003; Gardner et al. 2005; McMillen and Robinson 2005; Roseboom et al. 2006; Kwong et al. 2007). Interestingly, in the sheep, the impact of poor maternal nutrition around the time of conception on glucose tolerance is different in the offspring of singleton and twin pregnancies in early postnatal life (Todd et al. 2009). We have recently reported that exposure to maternal undernutrition before and during the first week after conception (periconceptional undernutrition; PCUN) resulted in a decrease in the protein abundance of key insulin signaling molecules in the skeletal muscle of singleton fetuses in late gestation (Lie et al. 2014). In twin fetuses exposed to PCUN, however, there was an increase in the protein abundance of key insulin signaling molecules. Similarly, in singleton and twin fetuses exposed to maternal undernutrition during the first week of pregnancy alone (i.e., preimplantation undernutrition; PIUN), there was an increase in the protein abundance of key insulin signaling molecules in fetal skeletal muscle. These findings suggest that in contrast to the PCUN singleton, that in the PIUN singleton and the PCUN and PIUN twin, there is a programming of an insulin-sensitive, rather than an insulin-resistant phenotype. We also note that neither PCUN nor PIUN resulted in any changes in body weight or crown rump length in the late gestation sheep fetus (Lie et al. 2014). The mechanisms by which exposure to undernutrition in early development result in the emergence of insulin-resistant or -sensitive phenotype in skeletal muscle may include programmed changes in pathways that regulate myogenesis and muscle growth and differentiation. It has been demonstrated, for example, that periconceptional or early gestational undernutrition in sheep resulted in a decrease in the total number of muscle fibers and altered muscle fiber composition, in singleton fetuses in late gestation (Quigley et al. 2005; Costello et al. 2008). A reduction in muscle fiber number could result from either a decrease in myoblast proliferation and differentiation or a decrease in protein synthesis in skeletal muscle during development, thus, decreasing muscle mass that can lead to insulin resistance and hyperglycemia in adult life.

**Figure 1:**
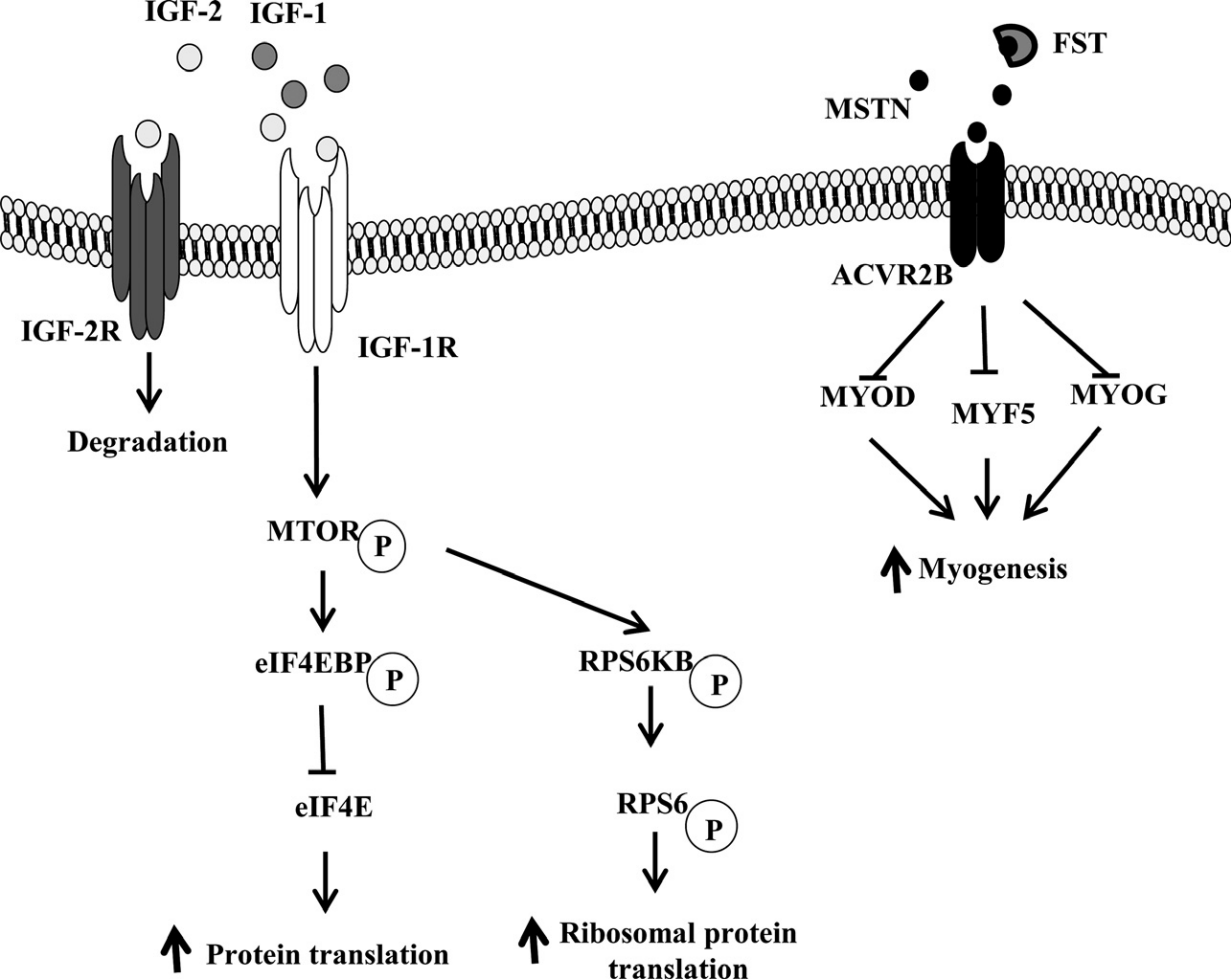
Molecular signaling pathways regulating protein translation, ribosomal protein translation, and myogenesis.

Insulin-like growth factor 1 and 2 (IGF1 and IGF2) promote myogenic differentiation through the insulin-like growth factor 1 receptor (IGF1R) or insulin receptor to activate the mechanistic target of rapamycin (MTOR) pathway (Duan et al. 2010) (Fig.1). Activation of MTOR, stimulates the release and activation of the eukaryotic translation initiation factor 4E (EIF4E) from the eukaryotic translation initiation factor 4E binding protein 1 (EIF4EBP1), thus initiating protein translation (Pause et al. 1994; Gingras et al. 1998), and activates ribosomal protein S6 kinase, 70 kDa (RPS6KB) (Brown et al. 1995) and ribosomal protein S6 (RPS6) which plays a role in the translation of mRNA encoding ribosomal protein (Kawasome et al. 1998). Therefore, the MTOR/EIF4E and MTOR/RPS6KB pathways regulate skeletal muscle protein synthesis and growth (Lang et al. 2010). IGF2 also binds to the insulin-like growth factor 2 receptor (IGF2R), which results in the lysosomal degradation of IGF2 (Kornfeld 1992).

Myostatin (MSTN) inhibits myoblast proliferation, differentiation, and protein synthesis through the downregulation of differentiation-related genes, such as myogenic differentiation (MYOD), myogenin (MYOG), and myogenic factor 5 (MYF5) (Grobet et al. 1997; McPherron and Lee 1997; Langley et al. 2002). The binding of MSTN to its receptor, the activin A receptor, type IIB (ACVR2B), is inhibited by follistatin (FST) (Lee et al. 2012), which blocks the inhibitory effect of MSTN on myogenesis (Amthor et al. 2004) (Fig.1). In the perinatal period, skeletal muscle is composed of myofibers expressing the myosin, heavy chain 8, skeletal muscle, perinatal (MYH8) (Young et al. 1994).

MicroRNAs have been shown to play a major role in mediating the programming effects of exposure to poor maternal nutrition before and during gestation (Herrera et al. 2010; Guay et al. 2011; Rottiers et al. 2011). In our previous study, we demonstrated that there were specific patterns of the types and direction of changes in the expression of 22 miRs in skeletal muscle after exposure to PCUN or PIUN and that there were clear differences in these patterns between singleton and twin pregnancies (Lie et al. 2014). A number of these miRs had identified targets in the insulin signaling pathway, for example, miR-126-5p, miR-106b-5p miR-126-5p, miR-21-5p, and miR-369-3p (Lie et al. 2014). Interestingly, a number of these miRs also had specific targets in the IGF signaling, myogenic, and protein synthesis pathways (as shown in Table S1).

In the current study, we have therefore investigated the separate effects of maternal undernutrition in the periconceptional period (PCUN: for at least 2 months before and 1 week after conception) or the preimplantation period (PIUN: for 1 week after conception) on the mRNA expression and protein abundance of factors involved in myogenesis and protein synthesis in the skeletal muscle of the sheep fetus in singleton and twin pregnancies. We hypothesized, based on our findings on the abundance of insulin signaling molecules in the fetal skeletal muscle of the PCUN and PIUN groups, that exposure to PCUN would result in a lower abundance of myogenic factors in singletons and a higher abundance of these factors in the skeletal muscle of twins. We also hypothesized that exposure to PIUN would result in a higher abundance of myogenic factors in skeletal muscle in both singleton and twin fetuses. Finally, we hypothesized that there would be a relationship between the level of expression of those candidate miRs which were changed after exposure to either PCUN or PIUN (Lie et al. 2014) and the abundance of regulatory factors in the IGF, myogenic, and protein synthesis pathways in fetal skeletal muscle.

## Materials and Methods

All procedures were approved by The University of Adelaide Animal Ethics Committee and by the Primary Industries and Resources South Australia Animal Ethics Committee (Lie et al. 2014; Williams-Wyss et al. 2014).

### Nutritional management

South Australian Merino ewes were fed a diet, which consisted of Lucerne chaff and pellets containing cereal hay, Lucerne hay, barley, oats, almond shells, lupins, oat bran, lime, and molasses (Johnsons & Sons Pty. Ltd., Kapunda, SA, Australia), as previously described (Lie et al. 2014; Williams-Wyss et al. 2014). All ewes received 100% of nutritional requirements to provide sufficient energy for the maintenance of a nonpregnant ewe as defined by the Agricultural and Food Research Council (Energy and Protein Requirements of Ruminants) in 1993.

At the end of an acclimatization period, ewes were randomly assigned to one of three feeding regimes as previously described (Lie et al. 2014). The Control ewes (C) (*n *=* *11) received 100% of the nutritional requirements from 84.5 ± 7.0 days prior to mating until 6 days after mating. Ewes in the PCUN group (*n *=* *13) received 70% of the control allowance from 76.8 ± 4.0 days prior to mating until 6 days after mating. All of the dietary components were reduced by an equal amount in the restricted diet. Ewes in the PIUN group (*n *=* *9) received 70% of the control diet from mating until 6 days after mating. All of the dietary components were reduced by an equal amount in the restricted diet. From 7 days after conception, all ewes were fed 100% of nutritional requirements.

### Mating, fetal outcomes, and postmortem

Ewes were released in a group every evening with rams of proven fertility that were fitted with harnesses and marker crayons. Ewes were individually housed the following morning and the occurrence of mating was confirmed by the presence of a crayon mark on the ewe’s rump. The day of mating was defined as day 0. Ewes were weighed weekly after commencing the feeding regime until postmortem at 135–138 days of gestation. The body weight of the ewes in the PCUN group was significantly lower compared to the body weight of the Control and PIUN groups during the preconceptional period (Williams-Wyss et al. 2014). Pregnancy and fetal number were estimated by ultrasound at 40–80 days of gestation and the nutritional intake for the ewes was adjusted for gestational age and fetal number.

All ewes carrying fetuses used in this study (*n *=* *33) were humanely killed with an overdose of sodium pentobarbitone between 135 and 138 days of gestation and the uteroplacental unit was delivered by hysterotomy. In four ewes carrying twin fetuses, fetal health declined prior to postmortem and/or a twin fetus died within 24 h of the postmortem, in these instances fetal tissues were not collected. Fetuses (Singleton: Controls *n *=* *5, PCUN *n *=* *8, PIUN *n *=* *3; Twin: Controls *n *=* *11, PCUN *n *=* *8, PIUN *n *=* *11) were weighed and killed by decapitation. Crown rump length and body weight was measured and samples were collected from the fetal quadriceps muscle (from the rectus femoris muscle bundle beneath the perimysium) and snap frozen in liquid nitrogen. Samples were then stored at −80°C for further molecular analyses. Details of the number of animals included in the study for the range of analyses are provided in Table1.

**Table 1:** Number of animals from each treatment group in singleton and twin pregnancies used in each set of analyses

	Singletons			Twins		
	Control	PCUN	PIUN	Control	PCUN	PIUN
Ewes	5	8	3	6	5	6
Fetal sheep	5	8	3	11	8	11
mRNA expression	5	8	3	11	8	11
Protein abundance	4	4	3	4	4	5
miR expression	3	3	3	3	3	3

PCUN, periconceptional undernutrition; PIUN, preimplantation undernutrition.

### Quantification of mRNA expression

RNA was extracted from ∼70 mg of quadriceps muscle tissue from singleton and twin fetuses (Table1) The relative expression of mRNA transcripts of *IGF1*, *IGF2*, *IGF1R*, *IGF2R*, *MTOR*, *RPS6KB*, *MSTN*, *FST*, *ACVR2B*, *MYOD*, *MYOG*, *MYF5*, *MYH8*, and the housekeeper gene peptidylprolylisomerase A (cyclophilin A; *PPIA*) was measured by quantitative real time reverse transcription PCR (qRT-PCR) using the Sybr Green system in an ABI Prism 7500 Sequence Detection System (Applied Biosystems, Foster City, CA), as previously described (Lie et al. 2014). Primer sequences were validated for use in the sheep in this study (Table2) or in prior studies (MacLaughlin et al. 2007).

**Table 2:** Primer sequences for qRT-PCR

Gene name	Sequence	Accession no.
PPIA	F: 5′ CCTGCTTTCACAGAATAATTCCA 3′	BC105173
	R: 5′ CATTTGCCATGGACAAGATGCCA 3′	
MTOR	F: 5′ TGACCATCCTCTGCCAACAGTTCA 3′	FJ617140.1
	R: 5′ GCTGCATGGTCTGAACAAAGTGCT 3′	
RPS6KB	F: 5′ ACTCAGCTCTCAGTGAAAGTGCCA 3′	NM_205816.1
	R: 5′ GGTGTTCGTGGGCTGCCAATAAAT 3′	
MSTN	F: 5′ TCGCCTGGAAACAGCTCCTAACAT 3′	NM_001009428
	R: 5′ ATCAGACTCCGTGGGCATGGTAAT 3′	
FST	F: 5′ TGCACTCCTCAAGGCCAGATGTAA 3′	M63123
	R: 5′ ATTAGTCTGGTCCACCACGCATGT 3′	
ACVR2B	F: 5′ TGCCCACAGGGACTTTAAGAGCAA 3′	AF420480.1
	R: 5′ GAAAGGCGTCTCTCTGGAAGTTGA 3′	
MYF5	F: 5′ ATGGCATGCCTGAATGTAACAGCC 3′	AF434668.1
	R: 5′ ATCCAGGTTGCTCTGAGTTGGTGA 3′	
MYOD	F: 5′ CTCAAACGCTGCACGTCTAGCAA 3′	NM_001009390.1
	R: 5′ GCCTTCGATATAGCGGATTGCGTT 3′	
MYOG	F: 5′ CTACAGATGCCCACAATCTGCACT 3′	NM_001174109.1
	R: 5′ TGGTATGGTTTCATCTGGGAAGGC 3′	
MYH8	F: 5′ AACGTGGAGCAACTCTCACTGTCA 3′	NM_001206174.1
	R: 5′ TGGCCATGTCCTCGATCTTGTCAT 3′	

The abundance of each mRNA transcript was measured and expression relative to *PPIA* was calculated using the comparative threshold cycle (C_*t*_) method (Q-gene qRT-PCR analysis software).

### Quantification of protein abundance

The protein abundance of IGF1R, IGF2R, MTOR, pMTOR (S2448), pMTOR (S2481), RPS6KB, pRPS6KB (T389), pEIF4EBP1 (T70), pEIF4EBP1 (S65), EIF4E, RPS6, pRPS6 (S235–236), MSTN, FST, ACVR2B, MYOD, MYOG, MYF5, and MYH8 were determined using western blotting, as previously described (Lie et al. 2014). Briefly, quadriceps muscle samples (∼200 mg) from singleton and twin fetuses (Table1) were homogenized in lysis buffer and protein content of the clarified extracts was quantified using bicinchoninic acid protein assay. Prior to western blot analysis, samples (10 *μ*g protein) were subjected to SDS-PAGE and stained with Coomassie blue reagent (Thermo Fisher Scientific, Rockford, IL) to ensure equal loading of the proteins. Equal volumes and concentrations of protein were subjected to SDS-PAGE. The membranes were blocked with 5% BSA in Tris-buffered saline with 0.1% Tween-20 (TBS-T) at room temperature for 1 h and then incubated overnight with primary antibody in TBS-T overnight at 4°C, against IGF1R, MTOR, pMTOR (S2448), pMTOR (S2481), RPS6KB, pRPS6KB (T389), pEIF4EBP1 (T70), pEIF4EBP1 (S65), EIF4E, RPS6, pRPS6 (S235–236) (1:1000 dilution; Cell Signalling, Danvers, MA), FST, MYH8 (1:200 dilution; Santa Cruz Biotechnology, Santa Cruz, CA), MYOD, MYOG, MYF5 (1:500 dilution; Epitomics, Burlingame, CA), MSTN, ACVR2B (1:500 dilution; Abcam, Cambridge, UK), and IGF2R (1:500 dilution; BD Transduction Laboratories, San Jose, CA). Membranes were washed and bound antibody detected using anti-rabbit or anti-mouse (Cell Signalling) or anti-goat (Merck Millipore, Billerica, MA) horseradish peroxidase-conjugated secondary IgG antibodies at room temperature for 1 h. Enhanced chemiluminescence reagents SuperSignal® West Pico Chemiluminescent Substrate (Thermo Fisher Scientific) and ImageQuant™ LAS 4000 (GE Healthcare, Rydalmere, NSW, Australia) was used to detect the protein:antibody complexes. AlphaEaseFC (Alpha Innotech Corporation, Santa Clara, CA) were utilized to quantify specific bands of the target proteins.

### Statistical analyses

#### mRNA expression and protein abundance

All data are presented as mean ± SEM. Data were analyzed using the Statistical Package for the Social Sciences Software (SPSS Inc., Chicago, IL). Two-way analysis of variance (ANOVA) was used to determine the effects of maternal nutritional treatment (PCUN, PIUN, or control) and fetal number (singleton or twin) on mRNA expression and protein abundance. When there was an interaction between the effects of nutritional treatment and fetal number, data from singletons and twins were split and the effects of nutritional treatment determined using a one way ANOVA. A Duncan’s post hoc test was used to determine the level of significant difference between mean values. A probability level of 5% (*P *<* *0.05) was taken as significant.

#### Relationship between miR expression and target protein abundance

We previously identified that the expression of a number of candidate miRs in fetal muscle was significantly altered by either PCUN and/or PIUN (Lie et al. 2014) using the following criteria: a threshold for a fold difference of expression of miRs between the PCUN or PIUN treatment groups relative to controls was set at >1.5 or <0.67 with a threshold of >1000 reads/million or at >1.2 or <0.83 with a threshold of >10,000 reads/million where the relative standard deviation was <50% among animals within a treatment group. Selected miRs based on these criteria from data mapped to the human miRBase were then cross checked with the corresponding miRs mapped to the bovine miRBase. miRs were then selected as high confidence “candidates.” Using the stringent threshold criteria defined in the methods, we identified 22 miRs with altered expression in either the PCUN or the PIUN groups relative to controls (Lie et al. 2014).

In the present study, these candidate miRs were analyzed using Targetscan to identify 8mer, 7mer-m8, or 7mer-1A matches between the seed sequence of the candidate miRs within the 3′ UTR of the putative mRNA targets within the IGF signaling, protein synthesis and myogenic pathways which are conserved across species (Table S1). The relationship between miR expression and the mRNA expression or protein abundance in muscle samples collected from the same animals was determined using linear regression analysis (SPSS Inc.).

## Results

### Impact of PCUN and PIUN on mRNA expression and protein abundance of the insulin-like growth factors in fetal skeletal muscle

**Figure 2:**
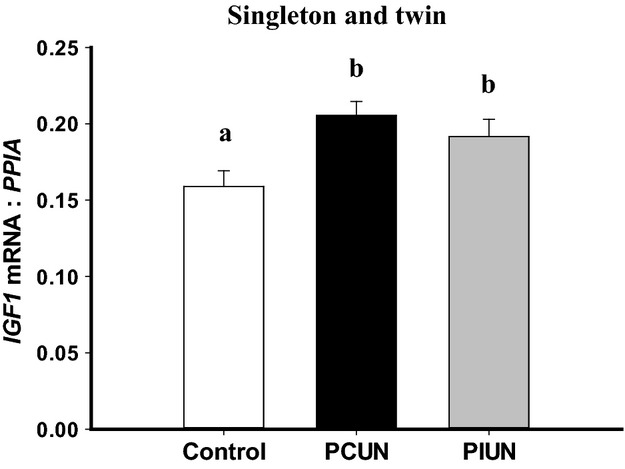
*IGF1* mRNA expression in the periconceptional undernutrition (PCUN) and preimplantation undernutrition (PIUN) groups compared to controls in singletons and twins. Different alphabetical subscripts denote significant differences between treatment groups compared to controls.

*IGF1* mRNA expression in fetal muscle was higher (*P *<* *0.01) in the PCUN and PIUN groups compared to controls in both singleton and twin pregnancies (Fig.2). There was no effect of either PCUN or PIUN, however, on *IGF2* mRNA expression (Table S2), or on the mRNA expression or protein abundance of IGF1R and IGF2R in fetal muscle in singletons or twins (Tables S2 and S3).

### Impact of PCUN and PIUN on mRNA expression and protein abundance of factors regulating protein synthesis in fetal skeletal muscle

#### Singletons

**Figure 3:**
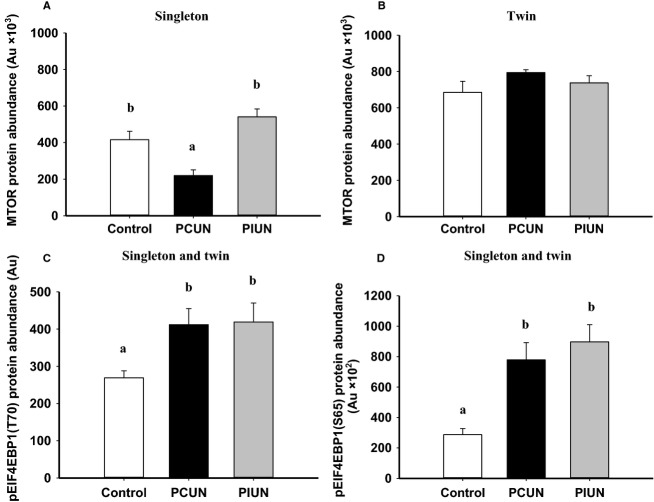
Protein abundance of mechanistic target of rapamycin (MTOR) in (A) singletons and (B) twins; protein abundance of phosphorylated EIF4EBP1 (T70) (C) and phosphorylated EIF4EBP1 (S65) (D) in singletons and twins in the periconceptional undernutrition (PCUN) and preimplantation undernutrition (PIUN) groups compared to controls. Different alphabetical subscripts denote significant differences between treatment groups compared to controls. Immunoblots are shown in Figure S1.

The protein abundance, but not mRNA expression of MTOR was lower (*P *<* *0.01) in muscle in the singleton PCUN fetal sheep compared to controls (Fig.3 and Table S2). There was no difference, however, in the abundance of phosphorylated MTOR (at either S2448 or S2481) between the PCUN, PIUN, and control groups (Table S3). The protein abundance of phosphorylated EIF4EBP1 (T70) (*P *<* *0.05) and phosphorylated EIF4EBP1(S65) (*P *<* *0.01) in singleton fetal muscle was higher, however, in both the PCUN and PIUN groups compared to controls (Fig.3).

**Figure 4:**
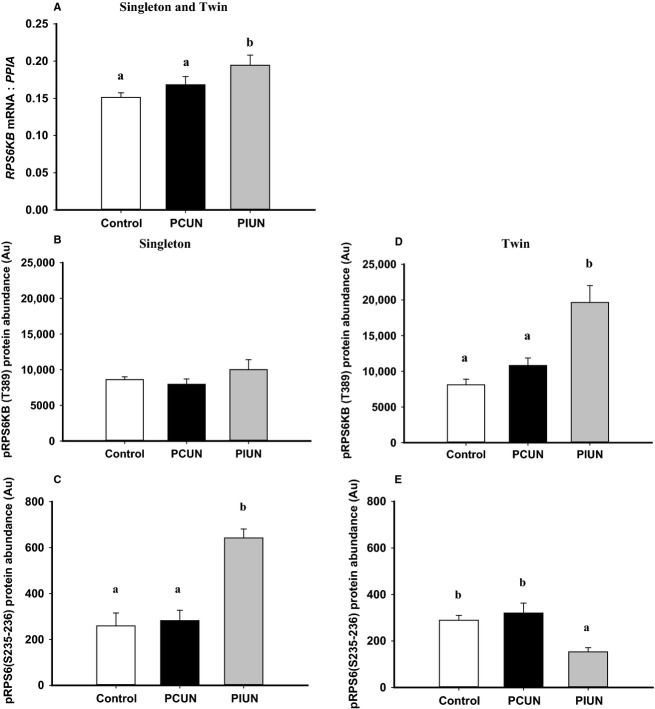
*RPS6KB* mRNA expression in singletons and twins in the PCUN and PIUN groups compared to controls (A). Protein abundance of phosphorylated RPS6KB (T389) (B) and phosphorylated RPS6 (S235-236) (C) in singletons in the PCUN and PIUN groups compared to controls. Protein abundance of phosphorylated RPS6KB (T389) (D) and phosphorylated RPS6 (S235-236) (E) in twins in the PCUN and PIUN groups compared to controls. Different alphabetical subscripts denote significant differences between treatment groups compared to controls. Immunoblots are shown in Supporting Figure S1.

The mRNA expression, but not the abundance of RPS6KB protein or the phosphorylated RPS6KB (T389) protein, was increased (*P *<* *0.05) in the PIUN group compared to controls in singletons (Fig.4 and Table S3).The protein abundance of phosphorylated RPS6 (S235–236) in fetal muscle was higher (*P* < 0.01) in the PIUN group compared to controls in singletons (Fig.4).

#### Twins

The protein abundance of MTOR in each of the control (*P *<* *0.05), PCUN (*P *<* *0.001), and PIUN (*P *<* *0.05) groups was higher in the skeletal muscle of twin compared to singleton fetuses (Fig.3). There was no difference in the mRNA expression or protein abundance of MTOR or phosphorylated MTOR at S2448 and S2481 (Tables S2 and S3) in fetal muscle between the PCUN, PIUN, and control groups. The protein abundance of phosphorylated EIF4EBP1 (T70) (*P *<* *0.05) and phosphorylated EIF4EBP1 (S65) (*P *<* *0.01) were each higher in twin fetal muscle in the PCUN and PIUN groups compared to controls (Fig.3). The mRNA expression, but not protein abundance of RPS6KB, was increased (*P *<* *0.05) in the PIUN group compared to controls in twins (Fig.4 and Table S3). The protein abundance of phosphorylated RPS6KB (T389) was higher (*P *<* *0.01), whereas the protein abundance of phosphorylated RPS6 (S235–236), was lower (*P *<* *0.01) in the twin fetal muscle of the PIUN group compared to controls (Fig.4).

### Impact of PCUN and PIUN on mRNA expression and protein abundance of factors regulating myogenesis in fetal skeletal muscle

#### Singletons and twins

**Figure 5:**
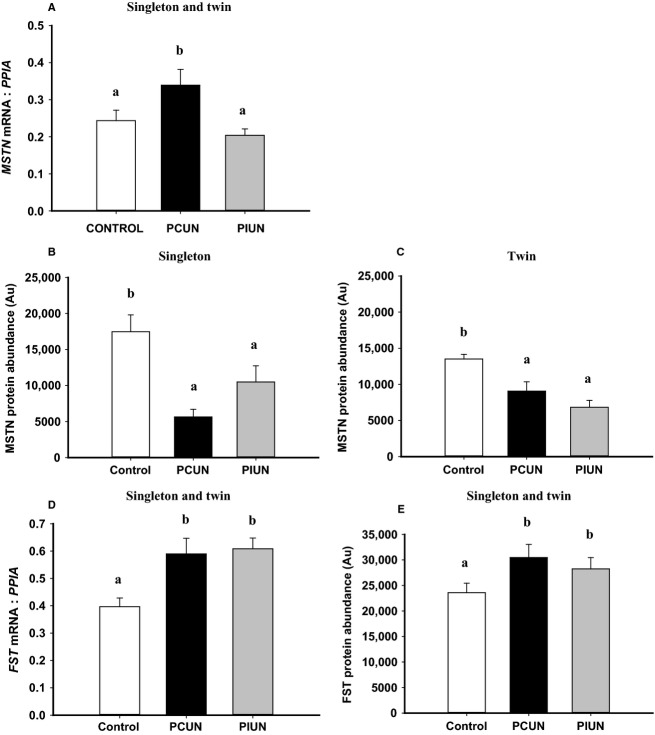
*MSTN* mRNA expression in singletons and twins (A), protein abundance of myostatin (MSTN) in singletons (B) and in twins (C), *FST* mRNA expression in singletons and twins (D), and protein abundance of follistatin (FST) in singletons and twins (E) in the periconceptional undernutrition (PCUN) and preimplantation undernutrition (PIUN) groups compared to controls. Different alphabetical subscripts denote significant differences between treatment groups compared to controls. Immunoblots are shown in Figure S1.

**Figure 6:**
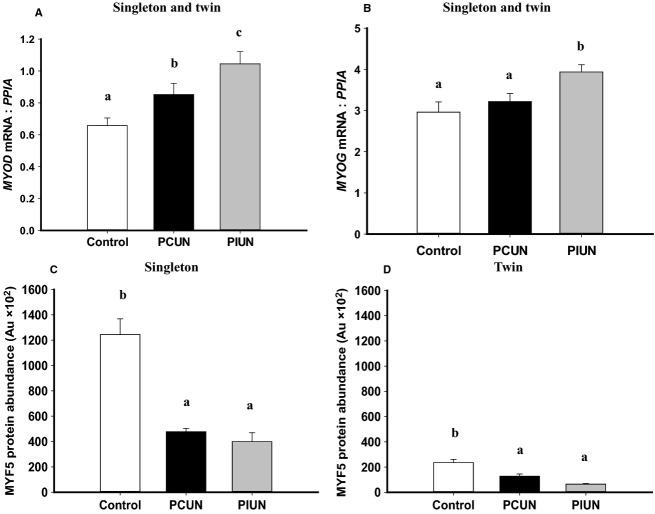
*MYOD* (A) and *MYOG* (B) mRNA expression in singletons and twins and protein abundance of MYF5 in singletons (C) and in twins (D) in the PCUN and PIUN groups compared to controls. Different alphabetical subscripts denote significant differences between treatment groups compared to controls in singletons and twins. Immunoblots are shown in Figure S1.

*MSTN* mRNA expression was higher (*P *<* *0.05) in fetal muscle in PCUN singletons and twins compared to controls, however, the protein abundance of MSTN was lower in PCUN and PIUN singleton (*P *<* *0.01) and twin (*P *<* *0.01) fetuses compared to controls (Fig.5). The mRNA (*P *<* *0.01) and protein abundance (*P *<* *0.01) of FST was higher in fetal muscle in PCUN and PIUN singleton and twin fetuses compared to controls (Fig.5). The mRNA expression of *MYOD* was higher (*P *<* *0.01) in the PCUN and PIUN groups, while the mRNA expression of *MYOG* was higher (*P *<* *0.05) in the PIUN group compared to controls, in singleton and twin fetuses (Fig.6). There was no difference, however, in the protein abundance of either MYOD or MYOG between the PCUN, PIUN, or control groups in singletons or twins (Table S3).

The protein abundance of MYF5 in each of the PCUN (*P *<* *0.001), PIUN (*P *<* *0.01), and control (*P *<* *0.001) groups was lower in twins compared to the protein abundance of MYF5 in singletons (Fig.6). MYF5 protein abundance, but not mRNA expression, was also lower in the fetal muscle of the PCUN and PIUN groups compared to controls in singletons (*P *<* *0.001) and twins (*P *<* *0.01) (Fig.6 and Table S2).

**Figure 7:**
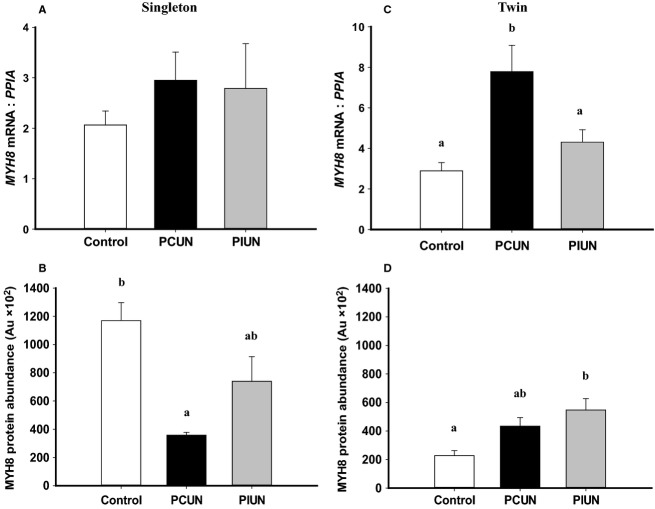
MYH8 mRNA expression (A) and protein abundance (B) in singletons and MYH8 mRNA expression (C) and protein abundance (D) in twins in the periconceptional undernutrition (PCUN) and preimplantation undernutrition (PIUN) groups compared to controls. Different alphabetical subscripts denote significant differences between treatment groups compared to controls. Immunoblots are shown in Figure S1.

The protein abundance of MYH8 in control twins was also lower (*P *<* *0.01) compared to control singletons (Fig.7). In singleton fetuses, the protein abundance, but not mRNA expression of MYH8 was lower (*P *<* *0.05) in the PCUN group compared to controls (Fig.7). In twins, however, the mRNA expression of *MYH8* was higher (*P *<* *0.05) in the PCUN group, while the protein abundance of MYH8 was higher (*P *<* *0.05) in the PIUN group compared to controls (Fig.7).

### Relationship between expression of specific miRs and the protein abundance of factors regulating protein synthesis and myogenesis

Using Targetscan software, we found that 17 of the 22 miRs that had altered expression in the fetal muscle of the PCUN or PIUN groups relative to controls were predicted to regulate the protein abundance of key factors within the protein synthesis or myogenic signaling pathways (Table S1).

In singletons, there was an inverse relationship between the expression of miR-30a-5p (*P *<* *0.05, *R*^2^ = 0.53) or the +1 isomir of miR-30d-5p (*P *<* *0.01, *R*^2^ = 0.71) and the protein abundance of MTOR; and between the +1 isomir of miR-106b-5p and the protein abundance of FST in fetal skeletal muscle (*P *<* *0.05, *R*^2^ = 0.59) (Table3).

**Table 3:** Relationship between the expression of candidate miRs and the protein abundance of the factors regulating myogenesis and protein synthesis in fetal skeletal muscle

microRNA	MTOR	FST	MYF5
hsa-miR-30a-5p	*y *=* *−91*x* + 815,475		*y *=* *−8*x* + 49,361
	*P *<* *0.05, *R*^2 ^= 0.53		*P *<* *0.01, *R*^2 ^= 0.84
	Singletons only		Twins only
hsa-miR-30d-5p (+1 isomir)	*y *=* *−69*x* + 874,941		
	*P *<* *0.01, *R*^2 ^= 0.71		
	Singletons only		
hsa-miR-27b-3p (+1 isomir)	*y *=* *−88*x* + 884,865		
	*P *<* *0.05, *R*^2 ^= 0.53		
	Twins only		
hsa-miR-106b-5p (+1 isomir)		*y *=* *−8*x* + 44,255	
		*P *<* *0.05, *R*^2^=0.59	
		Singletons only	
hsa-miR-376b (+1 isomir)			*y *=* *−20*x* + 32,191
			*P *<* *0.05, *R*^2 ^= 0.54
			Twins only

hsa denotes that the data were mapped to human miRBase.

MTOR, mechanistic target of rapamycin; FST, follistatin.

In twins, there was an inverse relationship between the expression of miR-30a-5p (*P *<* *0.01, *R*^2^ = 0.84) or the +1 isomir of miR-376b (*P *<* *0.05, *R*^2^ = 0.54) and the protein abundance of MYF5, as well as between the expression of the +1 isomir of miR-27b-3p and the protein abundance of MTOR (*P *<* *0.05, *R*^2^ = 0.53) (Table3).

## Discussion

We have demonstrated that maternal undernutrition during the periconceptional and/or preimplantation periods results in significant changes in the mRNA expression and/or protein abundance of factors regulating myogenesis and protein synthesis in the fetal quadriceps muscle and that these effects are different in singletons and twins in late gestation. We have also demonstrated that in a number of instances where PCUN or PIUN resulted in changes in the abundance of specific molecules regulating protein synthesis and myogenesis in skeletal muscle, there were significant relationships between the abundance of these molecules and the level of expression of specific miRs in the fetal muscle.

### Impact of PCUN and PIUN on the mRNA expression and protein abundance of factors regulating myogenesis

The protein abundance of the myogenic inhibitor, MSTN, was decreased in the PCUN and PIUN groups in both singleton and twin pregnancies. Additionally, the mRNA expression and protein abundance of the MSTN inhibitor, FST, was increased in the PCUN and PIUN groups in singletons and twins. Interestingly there was an associated increase in the *MYOD* mRNA expression in muscle in the PCUN and PIUN fetal sheep and an increase in the *MYOG* mRNA expression in PIUN fetuses, although these changes were not associated with an increase in MYOD and MYOG protein abundance. The protein abundance of MYF5, a factor that has been shown to regulate determination of the myogenic lineage (Megeney and Rudnicki 1995; Perry and Rudnick 2000), was decreased, however, in the fetal muscle in PCUN and PIUN singletons and twins. It is possible that the epigenetic profile of MYF5 was altered in response to poor nutrition during the periconceptional or preimplantation period, resulting in altered mRNA expression that persist at least into the late gestation. Consistent with this is the decrease in total muscle fiber number that has been reported in singleton fetal sheep at 75 days gestation, following PCUN from 18 days before to 6 days after conception in sheep (Quigley et al. 2005). However, nutritional environment of these fetuses has been normalized from 7 days postconception. Therefore, the decrease in MSTN and increased FST may be a compensatory response to a decrease in MYF5 abundance evoked by the impact of poor maternal nutrition on the embryo in the period immediately after conception, which may result in no net effect of muscle mass, contrary to findings from previous studies (Quigley et al. 2005; Costello et al. 2008). These compensatory responses may be mediated by an increase in protein translation, discussed later. Furthermore, MSTN and FST are expressed in the embryo in different species including zebrafish (Bauer et al. 1998; Vianello et al. 2003), mouse (Albano and Smith 1994; McPherron et al. 1997), and cattle (Kambadur et al. 1997; Yoshioka et al. 1998) and MSTN was recently shown to inhibit glucose uptake and consumption in mice skeletal muscle cells (Chen et al. 2010). It is therefore possible that maternal nutrient restriction during oocyte maturation and/or the preimplantation period results in the programming of an increase in energy production, again ensuring survival and appropriate development of the skeletal muscle.

Additionally, MYF5 protein abundance was lower in the skeletal muscle of twin compared to singleton fetuses which suggests that there may also be a smaller pool of cells destined for the myogenic cell lineage resulting in a lower myofiber number in twins. This may be a mechanism initiated in the twin embryo from conception or alternatively it may be a response within the developing skeletal muscle to the lower nutritional environment of a twin pregnancy. In either instance such a response would act to limit muscle mass and hence fetal growth in the twin pregnancy (Alexander et al. 1998; Joseph KS et al. 2003).

MYH8 abundance was lower in the fetal muscle in PCUN singletons and higher in PIUN twins. One possibility is that in the PCUN singleton, the decrease in MYH8 is a consequence of a decrease in total myofiber number due to the decrease in MYF5 abundance. In the PIUN groups, the decrease in MYF5 may be compensated for by the increase in EIF4EBP1 and RPS6 phosphorylation (as discussed later) and an associated increase in protein synthesis.

### Impact of PCUN and PIUN on the mRNA expression and protein abundance of factors regulating protein synthesis in fetal skeletal muscle

There was an increase in the mRNA expression of *IGF1*, which may promote myogenic differentiation (Duan et al. 2010) and/or protein synthesis through the activation of MTOR (Pause et al. 1994; Gingras et al. 1998) in the fetal muscle in the PCUN and PIUN groups compared to controls. Despite the increase in *IGF1* mRNA, however, there was no difference in the protein abundance of phosphorylated MTOR (S2448 and S2481). The phosphorylation of EIF4EBP1 at T70 and S65, however, was increased in fetal muscle in the PCUN and PIUN groups in both singletons and twins. This increase in EIF4EBP1 phosphorylation and the associated increase in the release of EIF4E from EIF4EBP1 may result in increased protein synthesis initiation (Pause et al. 1994; Gingras et al. 1998). One possibility is that PCUN and PIUN act to alter the abundance and/or activation of kinases known to regulate the phosphorylation of EIF4EBP1, namely cyclin-dependent kinase 1 (CDK1), protein kinase C, alpha (PRKCA), or calcium/calmodulin-dependent protein kinase kinase2, beta (CAMKK2) (Pons et al. 2012). We also found that in singletons, there was no change in the protein abundance of phosphorylated RPS6KB (T389), but an increase in the protein abundance of phosphorylated RPS6 (S235–236) in the PIUN group. In twins, however, there was an increase in the protein abundance of phosphorylated RPS6KB (T389), but a paradoxical decrease in the protein abundance of phosphorylated RPS6 (S235–236), also in the PIUN group. RPS6KB has been shown to regulate the phosphorylation of RPS6 (Kawasome et al. 1998), therefore there is an apparent disconnect between the activation of RPS6KB and the phosphorylation of RPS6 (S235 and S236). More recent studies, however, have shown that RPS6KB is dispensable for the phosphorylation of RPS6 at S235 and S236 (Roux et al. 2007), and that the RAS/extracellular signal-regulated kinases (ERK) pathway is crucial for the phosphorylation of RPS6 at S235 and S236 through the activation of ribosomal protein S6 kinase, 90 kDa (RPS6KBA) (Pende et al. 2004; Roux et al. 2007). Therefore, PIUN may alter the RAS/ERK pathway differently in singletons and twins, which may explain the changes present in the phosphorylation of RPS6 (S235 and S236) in this study.

### Role of specific miRs in regulating the abundance of key factors in muscle growth and development in fetal skeletal muscle

In a previous study, we identified 22 miRs with altered expression in skeletal muscle in the PCUN and PIUN groups (Lie et al. 2014). In this study, we have shown that 17 of the 22 miRs were predicted to regulate the protein abundance of key factors regulating myogenesis and protein synthesis (Table S1). We also found that the expression of five miRs was significantly related to the abundance of three key proteins that regulate protein synthesis and myogenesis (Table3). In particular, the expression of miR-30a-5p was increased and the abundance of MYF5 was decreased in PCUN and PIUN twins which may support the conclusion that the impact of PCUN and PIUN is predominantly on the embryo in the period immediately post conception (Table S1).

A number of the miRs which we identified in our previous study have altered expression in response to PCUN or PIUN (Lie et al. 2014). These include miR-126-5p, miR-30a-5p, and miR-30d (Guay et al. 2011), as well as miR-27b, miR-21, miR-206 (Herrera et al. 2010), and let-7 family (Frost and Olson 2011), each of which is altered in the states of insulin resistance, glucose intolerance, and/or type-2 diabetes in adult life. Also, it is interesting that in our model, PIUN resulted in increased expression of miR-206 in twin fetal skeletal muscle. miR-206 has been shown to have a negative correlation with daily physical activity and may contribute to chronic obstructive pulmonary disease associated skeletal muscle dysfunction (Lewis et al. 2008) and its expression is decreased in a mouse model of Duchenne muscular dystrophy (McCarthy et al. 2007).

In summary, in both singletons and twins, PCUN and PIUN result in an increased *IGF1* mRNA expression and a decrease in the abundance of MSTN protein and an increase in the MSTN inhibitor, FST mRNA, and protein in fetal skeletal muscle. We suggest that these changes are in response to the decrease in the protein abundance of MYF5 in singletons and twins in the PCUN and PIUN groups (Quigley et al. 2005; Costello et al. 2008), and that this may be the dominant contributor to the decrease in total myofiber number and to the emergence of whole body insulin resistance in postnatal life. PCUN and PIUN also increased the abundance of phosphorylated EIF4EBP1 (T70 and S65) in both singletons and twins, which may result in an increase in protein synthesis, perhaps as a compensatory response to maintain skeletal muscle mass particularly when fetal nutrition is adequate. Interestingly, PIUN resulted in an increase in phosphorylated RPS6 (S235–236) in singletons, but a decrease in PIUN twins, independent of RPS6KB activation. Therefore, PIUN may alter other pathways, namely the RAS/ERK pathway, to play a role in regulating mitochondrial biogenesis and thus protein synthesis in skeletal muscle.

We have previously reported that PCUN decreased the abundance of key insulin signaling molecules in fetal muscle in singletons, while PIUN in singletons as well as PCUN and PIUN in twins resulted in an increased abundance of a different subset of insulin signaling molecules in skeletal muscle (Lie et al. 2014). Therefore, decreased myogenesis coupled with a decrease in key insulin signaling molecules in PCUN singleton fetuses may result in the increased risk of insulin resistance and impaired glucose uptake which occurs in response to PCUN in later life. In singletons exposed to PIUN and in twins exposed to PCUN and PIUN, however, the potential decrease in myogenesis may be compensated for by an increased protein synthesis which may maintain muscle mass in these groups.

We have also shown that PCUN and/or PIUN result in altered expression of specific miRs that regulate myogenesis and protein synthesis, which suggest that the impact of PCUN and PIUN is predominantly on the embryo during early embryogenesis.

Findings from this study provides evidence that poor maternal nutrition during the periconceptional period alone is sufficient to result in programmed changes in the key factors known to regulate muscle growth and development, and thus highlights the importance of adequate maternal nutrition before and during early embryonic development. However, the impact of these changes in postnatal life and thus their contribution to metabolic dysfunction, namely insulin resistance and glucose intolerance, will require further investigation.

## Supporting Information

**Figure S1.** Immunoblots of proteins regulating skeletal muscle growth and development in singletons and twins in fetal skeletal muscle.

**Table S1.** Impact of PCUN and PIUN on the expression of candidate miRs in singletons and twins in fetal skeletal muscle (Lie et al. 2014) and the predicted target proteins within the myogenesis and protein synthesis pathwayin fetal skeletal muscle.

**Table S2.** Impact of PCUN and PIUN on mRNA expression of factors regulating skeletal muscle growth and development in singletons and twins in fetal skeletal muscle.

**Table S3.** Impact of PCUN and PIUN on protein abundance of factors regulating skeletal muscle growth and development in singletons and twins in fetal skeletal muscle.
