# Supplementary material for: Impact of periconceptional and preimplantation undernutrition on factors regulating myogenesis and protein synthesis in muscle of singleton and twin fetal sheep
Source: Physiol Rep. 2015 Aug 11;3(8):e12495. doi: 10.14814/phy2.12495 (PMC4562581; doi:10.14814/phy2.12495)

**Supporting Figure 1**

**Skeletal muscle MTOR protein bands**

**
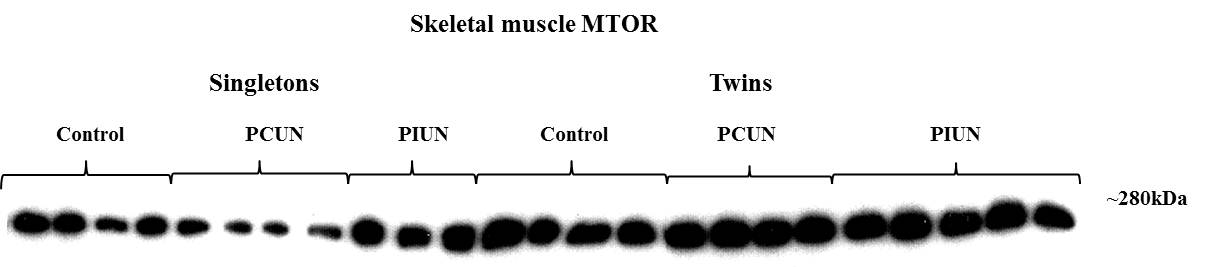
**

**Skeletal muscle pEIF4EBP1 (T70) protein bands**

**
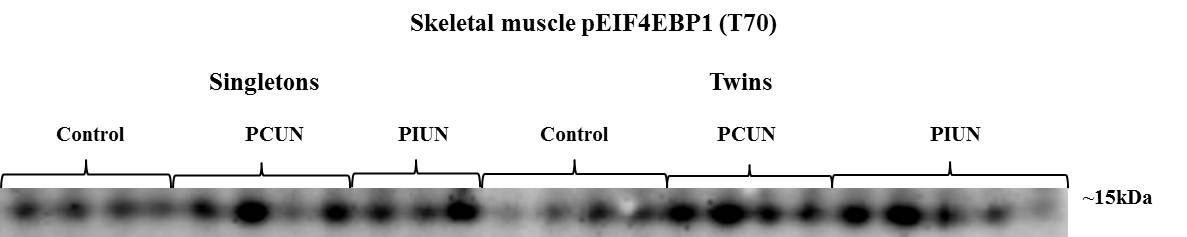
**

**Skeletal muscle pEIF4EBP1 (S65) protein bands**

**
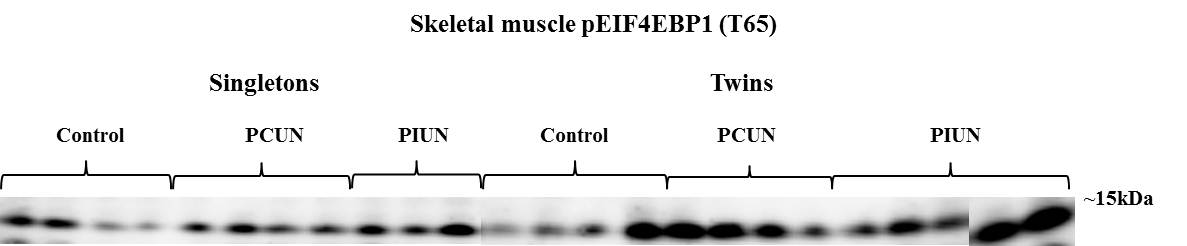
**

**Skeletal muscle pRPS6KB (T389) protein bands**

**
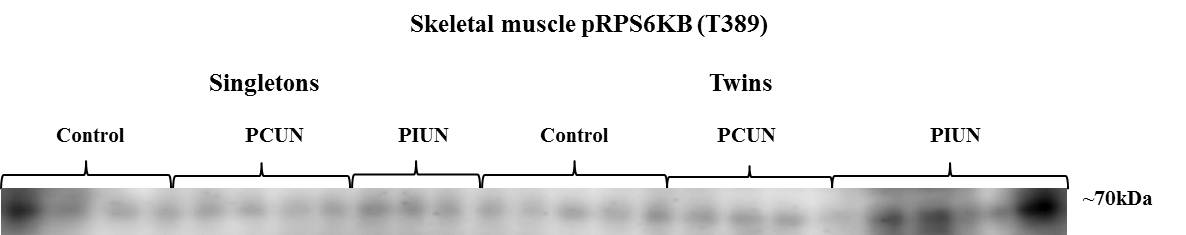
**

**Skeletal muscle pRPS6 (S235-236) protein bands**

**
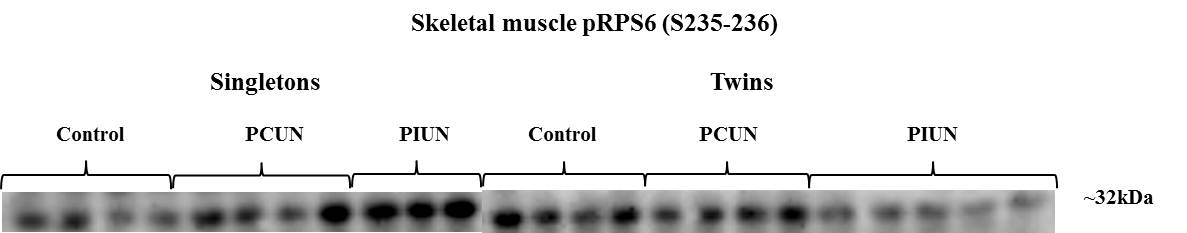
**

**Skeletal muscle MSTN protein bands**

**
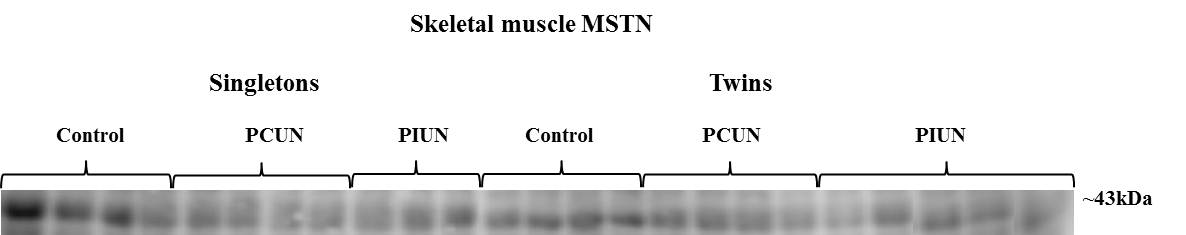
**

**Skeletal muscle FST protein bands**

**
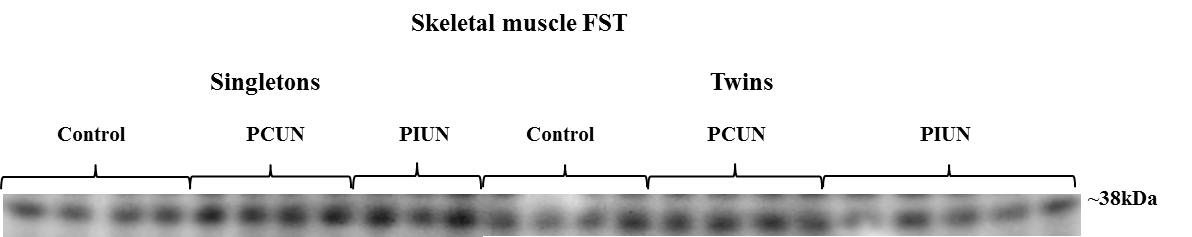
**

**Skeletal muscle MYF5 protein bands**

**
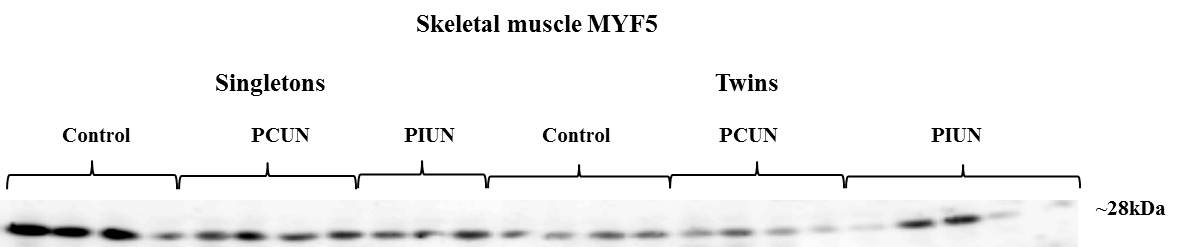
**

**Skeletal muscle MYH8 protein bands**


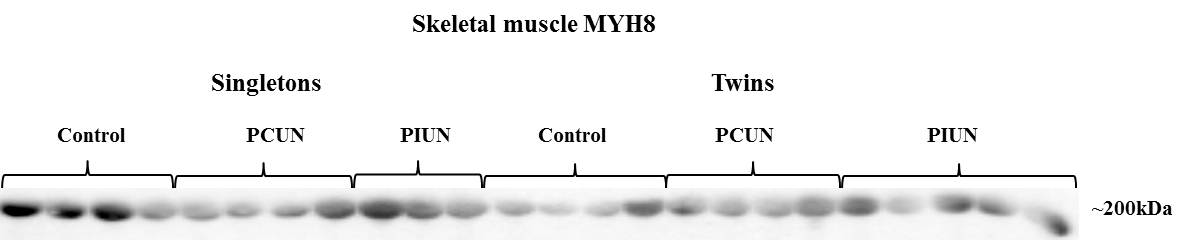

Supplement: Supplementary file 1 [file phy20003-e12495-sd1.docx]
