# Supplementary material for: Impact of periconceptional and preimplantation undernutrition on factors regulating myogenesis and protein synthesis in muscle of singleton and twin fetal sheep
Source: Physiol Rep. 2015 Aug 11;3(8):e12495. doi: 10.14814/phy2.12495 (PMC4562581; doi:10.14814/phy2.12495)
Supplement: Supplementary file 2 [file phy20003-e12495-sd2.docx]

**Supporting Table 1. Impact of PCUN and PIUN on the expression of candidate miRs in singletons and twins in fetal skeletal muscle[**[**7**](#_ENREF_7)**]and the predicted target proteins within the myogenesis and protein synthesis pathwayin fetal skeletal muscle.**

|  | **Singleton Fetal Sheep** | | **Twin Fetal Sheep** | |  |  |  |
| --- | --- | --- | --- | --- | --- | --- | --- |
|  | **PCUN** | **PIUN** | **PCUN** | **PIUN** |  |  |  |
| **microRNA** | **Fold change relative to singleton controls** | **Fold change relative to singleton controls** | **Fold change relative to twin controls** | **Fold change relative to twin controls** | **Predicted target protein** | **nt match** | **No. of matches** |
| **hsa-miR-199b-3p** | **x1.66 ↑** |  |  | **x1.28 ↑** | **ACVR2B** | **8mer** | **1** |
| **hsa-miR-126-5p (+1 isomir)** | **x0.50 ↓** | **x0.64 ↓** |  |  | **IGF1** | **7mer-1A** | **1** |
| **hsa-miR-19a-3p (+1 isomir)** | **x0.45 ↓** | **x0.66 ↓** |  |  | **ACVR2B**  **IGF1**  **IGF1R**  **IGF2R** | **7mer-m8**  **7mer-m8**  **7mer-m8**  **7mer-m8** | **1**  **1**  **1**  **1** |
| **hsa-miR-381**  **(+1 isomir)** | **x0.31 ↓** | **x0.57 ↓** |  |  | **ACVR2B** | **8mer; 7mer-m8** | **1; 1** |
| **hsa-let-7i-5p**  **(+1 isomir)** | **x0.50 ↓** |  |  |  | **IGF1R**  **IGF1**  **ACVR2B** | **8mer; 7mer-m8; 7mer-1A**  **8mer**  **7mer-1A** | **1; 1; 1**  **1**  **2** |
| **hsa-miR-125a-5p (+1 isomir)** | **x0.67 ↓** |  |  |  | **ACVR2B** | **7mer-m8** | **1** |
| **hsa-miR-27b-3p (+1 isomir)** | **x0.64 ↓** |  |  |  | **IGF1** | **7mer-m8** | **1** |
| **hsa-let-7g-5p**  **(+1 isomir)** | **x0.50 ↓** |  |  |  | **IGF1** | **7mer-m8** | **1** |
| **hsa-miR-376b (+1 isomir)** | **x0.66 ↓** |  |  |  | **IGF1R** | **7mer-1A** | **1** |
| **hsa-miR-30a-5p** |  |  | **x1.62 ↑** | **x1.64 ↑** | **IGF2R**  **IGF1R**  **IGF1** | **8mer**  **7mer-1A**  **7mer-1A** | **1**  **1**  **1** |
| **hsa-miR-30d-5p (+1 isomir)** |  |  | **x1.52 ↑** |  | **ACVR2B**  **IGF1** | **8mer**  **7mer-1A** | **1**  **1** |
| **hsa-miR-21-5p (+1 isomir)** |  |  |  | **x1.22 ↑** | **IGF1** | **7mer-1A** | **1** |
| **hsa-miR-206**  **(+1 isomir)** |  |  |  | **x1.35 ↑** | **EIF4E** | **7mer-m8** | **1** |
| **hsa-miR-30e-5p** |  |  |  | **x1.53 ↑** | **IGF2R**  **IGF1R**  **IGF1** | **8mer**  **7mer-1A**  **7mer-1A** | **1**  **1**  **1** |
| **hsa-miR-26b-5p (+1 isomir)** |  |  | **x0.61 ↓** |  | **IGF1** | **7mer-1A** | **1** |
| **hsa-miR-369-3p (+1 isomir)** |  |  | **x0.49 ↓** | **x0.49 ↓** | **RPS6KB**  **EIF4E** | **7mer-1A**  **7mer-m8** | **1**  **1** |
| **hsa-miR-411-3p (+1 isomir)** |  |  |  | **x0.58 ↓** | **IGF1R**  **ACVR2B** | **7mer-m8**  **7mer-m8** | **2**  **1** |

**↑Fold change >1.5 or ↓fold change <0.67 with threshold of >1000 reads/million or ↑fold change >1.2 or ↓fold change <0.83 with threshold of >10,000 reads/million. hsa- denotes that the data were mapped to human miRBase.**
