# Supplementary material for: Impact of periconceptional and preimplantation undernutrition on factors regulating myogenesis and protein synthesis in muscle of singleton and twin fetal sheep
Source: Physiol Rep. 2015 Aug 11;3(8):e12495. doi: 10.14814/phy2.12495 (PMC4562581; doi:10.14814/phy2.12495)
Supplement: Supplementary file 3 [file phy20003-e12495-sd3.docx]

**Supporting Table 2. Impact of PCUN and PIUN on mRNA expression of factors regulating skeletal muscle growth and development in singletons and twins in fetal skeletal muscle**

|  | | **Target gene mRNA expression relative to *PPIA* mRNA expression** | | |
| --- | --- | --- | --- | --- |
| **Gene** | **Control** | **PCUN** | **PIUN** | |
| ***IGF2*** | 4.540 ± 0.227 | 4.795 ± 0.338 | 5.069 ± 0.368 | |
| ***IGF1R*** | 0.100 ± 0.005 | 0.093 ± 0.004 | 0.122 ± 0.012 | |
| ***IGF2R*** | 0.151 ± 0.009 | 0.161 ± 0.008 | 0.167 ± 0.009 | |
| ***MTOR*** | 0.019 ± 0.001 | 0.019 ± 0.001 | 0.021 ± 0.002 | |
| ***ACVR2B*** | 0.277 ± 0.019 | 0.284 ± 0.027 | 0.327 ± 0.026 | |
| ***MYF5*** | 0.121 ± 0.011 | 0.135 ± 0.015 | 0.167 ± 0.016 | |

**Data presented as mean ± standard error of mean.**
