# Supplementary material for: Impact of periconceptional and preimplantation undernutrition on factors regulating myogenesis and protein synthesis in muscle of singleton and twin fetal sheep
Source: Physiol Rep. 2015 Aug 11;3(8):e12495. doi: 10.14814/phy2.12495 (PMC4562581; doi:10.14814/phy2.12495)
Supplement: Supplementary file 4 [file phy20003-e12495-sd4.docx]

**Supporting Table 3. Impact of PCUN and PIUN on protein abundance of factors regulating skeletal muscle growth and development in singletons and twins in fetal skeletal muscle**

|  | **Protein abundance (Au x 10**^2^**)** | | |
| --- | --- | --- | --- |
| **Protein** | **Control** | **PCUN** | **PIUN** |
| **IGF1R** | 302 ± 31 | 240± 51 | 287± 50 |
| **IGF2R** | 42 ± 4 | 39± 5 | 34± 5 |
| **pMTOR (S2448)** | 3570 ± 259 | 3506± 368 | 4030± 314 |
| **pMTOR (S2481)** | 3584 ± 254 | 4602± 217 | 3805± 427 |
| **RPS6KB** | 7098 ± 568 | 7554± 812 | 6404± 836 |
| **ACVR2B** | 25116 ± 3874 | 24795 ± 2058 | 25479 ± 1861 |
| **MYOD** | 287 ± 45 | 354± 42 | 447± 39 |
| **MYOG** | 320 ± 18 | 383± 29 | 341± 21 |

**Data presented as mean ± standard error of mean.**
